# A Descriptive Study of Hot Aches: a Previously Unreported Winter Climbing Phenomenon

**DOI:** 10.1186/s40798-016-0062-z

**Published:** 2016-09-12

**Authors:** Andrew Melvin, Jacob George

**Affiliations:** Clinical Pharmacology and Therapeutics, Division of Molecular and Clinical Medicine, University of Dundee School of Medicine, Dundee, Scotland

**Keywords:** Vasospasm, Climbing, Mountaineering, Reactive hyperaemia, Ice climbing, Screaming barfies

## Abstract

**Background:**

Hot aches, also known as the screaming barfies in North America, are a recognised phenomenon amongst winter climbers, assumed to be triggered by the reperfusion of cold peripheries which then rapidly progresses to a systemic vasodilatory syndrome. Symptoms experienced in the hands include pain, numbness and throbbing followed by systemic symptoms such as nausea, irritability, dizziness and in extreme cases a transient loss of vision and hearing. Despite being well known amongst the winter climbing community, there are no publications in the scientific literature characterising the hot aches.

**Methods:**

A survey was posted online at http://www.ukclimbing.com between the dates of 28th September 2014 to 1st December 2014. Data was collected and analysed offline using Microsoft excel.

**Results:**

This is a descriptive epidemiological study of UK winter climbers and their experience of hot aches. We found that hot aches are experienced by 96 % of these climbers. They generally last 1–5 min, and 75 % rate them as being 3–4 (out of 5) on a pain scale. The most common local symptoms are pain (87 %), throbbing (70 %) and tingling (52 %). The most common systemic symptoms are nausea (44 %), irritability (32 %) and dizziness (20 %). Twenty percent of climbers experience hot aches in locations other than their hands.

**Conclusions:**

The hot aches are a highly predictable and consistent experience for almost all winter climbers. This study has characterised, for the first time, a recognised but previously unreported phenomenon that occurs in extreme winter climbers. The short- and long-term consequences are currently unknown and warrant further investigation.

**Electronic supplementary material:**

The online version of this article (doi:10.1186/s40798-016-0062-z) contains supplementary material, which is available to authorized users.

## Key Points

- The hot aches is a highly predictable syndrome lasting 1–5 min occurring in winter climbers thought to be due to reperfusion and systemic vasodilatation in response to cold exposure.
- Common local symptoms of hot aches include pain, throbbing and tingling which can progress to systemic symptoms including nausea, dizziness, transient loss of vision and irritability.
- The exact pathophysiology of this condition is unknown. We characterise, for the first time, the features associated with hot aches in this large descriptive study of winter climbers.

## Background

Winter climbing in the UK, predominantly in Scotland, is a popular sport during the months of November to April. It involves climbing ice, rock or steep snow in the mountains, usually in very inhospitable conditions using equipment that includes ice axes for hands and crampons for feet. Climbs are graded using an open-ended system agreed on by the climbing community that ranges from I, the easiest, to XII, the hardest. This grade takes into account many aspects of the climb including the physical difficulty and safety.

**Fig. 1:**
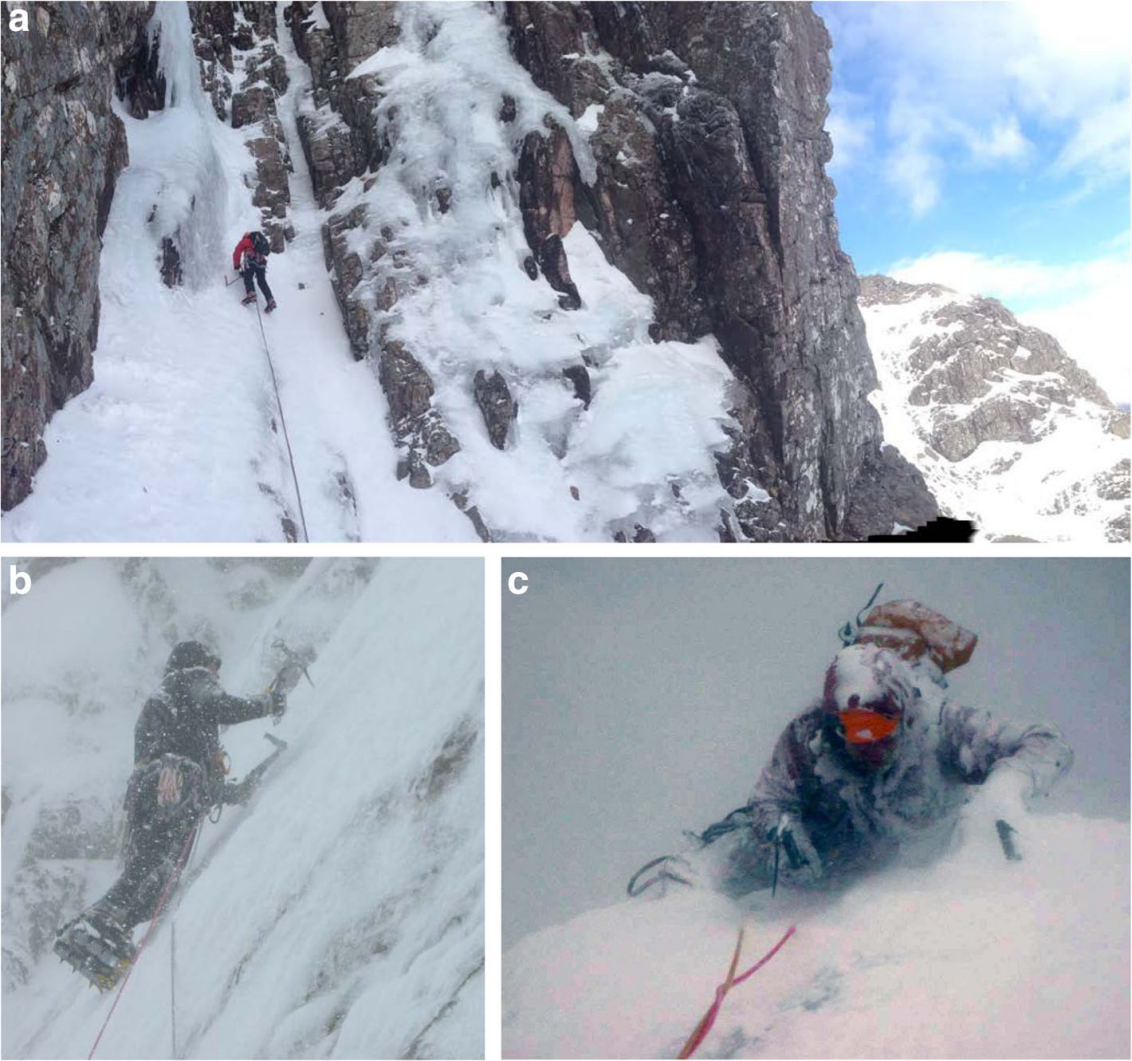
Photos of winter climbing in Scotland: **a** Boomer’s Requiem, Ben Nevis. **b** Hadrian’s Wall, Ben Nevis. **c** Smith’s route, Ben Nevis. All photos taken by Andrew Melvin

Winter climbing poses additional challenges, as the environment is, by definition, extremely cold (Fig. 1). The cold affects dexterity, concentration and performance, and continued exposure can lead to serious tissue damage such as frostbite [1, 2]. Normally, only one climber of a pair moves at a time; the second climber remains stationary, belaying the leading climber. Significant periods of immobility in sub-zero temperatures cause substantial loss of body heat and potential cold stress [3].

The hands rewarm either whilst climbing, once the body temperature has risen due to exertion or once the hands are no longer raised overhead or in contact with snow. The hot aches, also known amongst North American ice climbers as the screaming barfies, usually start when one lowers the hands, beginning initially with a throb that continues to increase in discomfort until reaching a climax, the symptoms of which are varied but include nausea and in extreme cases pinhole vision, muffled hearing and vomiting. The hot aches are notorious amongst climbers, but there is no documented evidence of their existence in the scientific literature. This study aims to define and characterise hot aches, by surveying the UK winter climbing community.

## Methods

The questionnaire was posted on http://www.ukclimbing.com between the dates of 28 September 2014 and 1 December 2014. The questionnaire was designed online using http://esurv.org, a free survey tool. Data was downloaded from esurv.org in Excel spreadsheet form.

The survey consisted of three sections. Section one contained personal details such as age, gender and medical history. Section two contained a climbing history where climbers were asked for their experience level and to report what grade of climber they considered themselves. UK winter climbing has its own grading system using Roman numerals from grade I to grade XII with the larger number representing overall difficulty and danger. Climbers constantly rate themselves on this scale and use their current grade to guide future performance [4]. Self-reporting has been shown to provide a valid and accurate reflection on climbing ability [4].

Finally, section three contained questions relating to hot aches such as onset, frequency, duration and associated symptoms.

Data analysis was performed in Microsoft Excel. Inclusion criteria: winter climbers. Exclusion criteria: incomplete survey (defined as failure to answer questions in part 2, regarding hot aches) and duplicate surveys from the same individual determined using IP address and information from part 1.

## Results

Four hundred eighty-seven people completed the online survey. Three surveys were excluded as duplicates, based on IP address and content. One hundred nineteen responders did not complete any questions relating to hot aches and were excluded. Three people failed to specify gender but were not excluded. Nine people did not specify ethnicity but were not excluded. Three hundred sixty-eight surveys were included.

Three hundred twenty-three (88.5 %) males and 42 (11.5 %) females completed the questionnaire, with the caveat that 3 did not specify gender. Thirty-two (8.7 %) climbers reported to be active smokers. All but two responders answered white as their ethnicity (357, 99.4 %), not including nine who withheld their ethnicity.

**Fig. 2:**
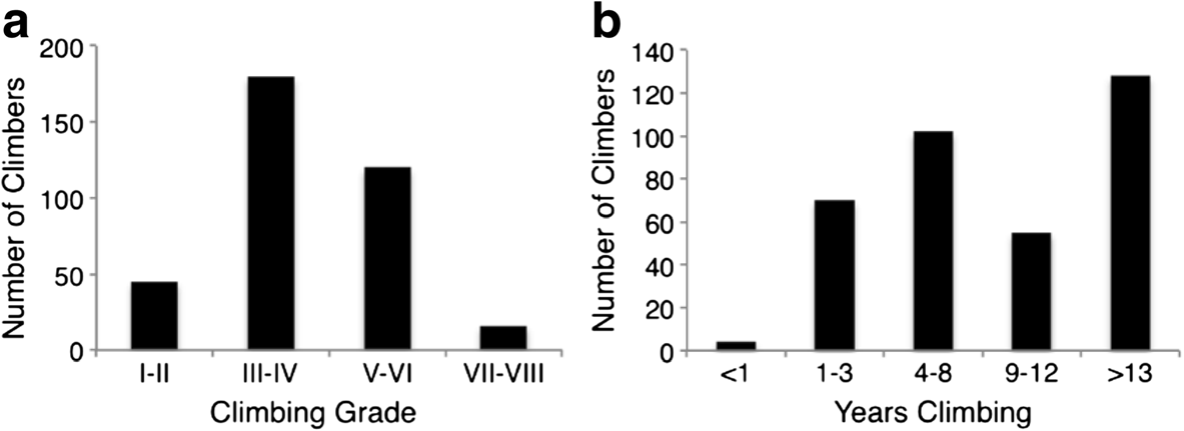
Survey results: **a** winter climbing grades climbed by responders; **b** number of years of climbing experience

The majority of climbers completing the survey climbed grades III to VI (Fig. 2a), with 45 (12.2 %) climbing easier grades and only 16 (4.3 %) climbers climbing harder. Figure 2b demonstrates the distribution of climbing experience amongst the responders, with the majority climbing for over 13 years.

**Fig. 3:**
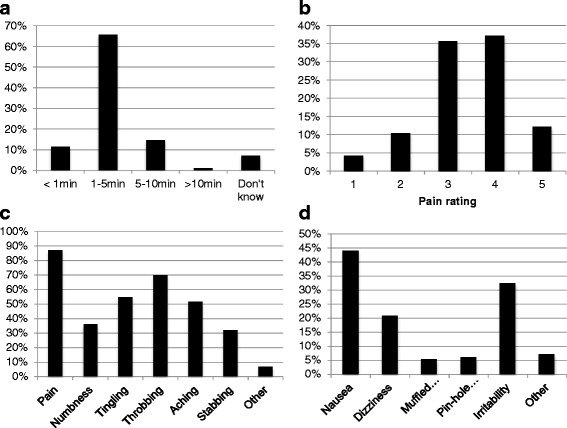
Survey results: **a** duration in minutes of hot aches; **b** abbreviated numerical pain rating scale (1 = not very, 5 = very severe); **c** local symptoms reported during hot aches; **d** systemic symptoms reported during hot aches

Hot aches are experienced by 96 % of winter climbers in our study. When asked to characterise the hot aches experience, 66 % of climbers reported them to last 1–5 min (Fig. 3a) and 75 % rating them as being 3–4 on the pain scale (rated 1–5) (Fig. 3b), with 5 being the highest rating. The hot aches are an unpleasant experience, and responders were asked to confirm symptoms that they experience both locally to the hands and also systemically. The most prevalent local symptom was pain, with 87 % of responders experiencing pain (Fig. 3c). Throbbing was the next most common sensation (70 %) followed by tingling (55 %) and aching (52 %). Nausea was the most common systemic symptom with 44 % of climbers experiencing nausea (Fig. 3d), although only 4 % of responders have ever actually vomited in response to getting the hot aches. Irritability is experienced by 32 % of responders and dizziness by 20 %. Interestingly, other features such as pinhole vision and muffled hearing are present but only experienced by 6 and 5 % of climbers, respectively. When asked if their experience of hot aches is fairly similar each time, 82 % of climbers responded that they are. Additionally, 20 % of climbers report getting hot aches in places other than their hands, with feet being the most common location.

In our study, 65 % of climbers experience hot aches at least once every three climbs, with the remainder experiencing them once a season or less. Hot aches are therefore a frequent event for those who winter climb. Sixty percent of climbers get hot aches once per climb and the remainder usually two or three times per climb (36 %). Finally, 27 % of climbers thought that they might be damaging their hands during the hot aches.

## Discussion

This study has characterised an undocumented phenomenon that occurs in winter climbers in response to cold exposure. There is a significant body of research that has investigated the response to [5, 6] and consequences [7, 8] of cold exposure, but none have documented this repeatable syndrome which occurs in winter climbers. There are several mechanisms that have been shown to play a role in response to cold exposure in the peripheries, but none have been directly linked to a hot ache syndrome [9]. These include cold-induced vascular dilatation (CIVD), a paradoxical cyclical increase in blood flow to cold limbs [10, 11], nonfreezing cold injury (NFCI) [12] and reactive hyperaemia [10].

A logical starting place is the regulators of the vascular endothelium such as nitric oxide, prostaglandins, adenosine triphosphate (ATP) channels and adenosine [10]. For example, there is in vivo evidence that the half life of nitric oxide (NO) may be in the range of seconds to minutes and that it may be transported considerable distances along the vascular bed [13]. If these factors persist long enough to enter systemic circulation, they may be responsible for causing the systemic symptoms of hot aches. Further research is required to investigate if any of these mechanisms are responsible.

One possibility for why hot aches have not been identified previously is that the cold stress and exertion experienced by climbers modify their response to cold [14]. This has been demonstrated with CIVD, which does not occur below a certain core temperature [15, 16]. It will be important to identify which conditions experienced by winter climbers increase the risk of the hot aches.

Finally, given the prevalence of hot aches amongst winter climbers, it would be of great interest to the community to identify precipitating and preventing factors. For example, does clothing [17], hydration [18], nutritional state [14] or diet [19, 20] play a role? Could the hot aches be prevented or reduced with medication such as glyceryl trinitrate (GTN) [12]? There are also implications for safety [5, 21] as climbers depended on dexterity for their safety. It is also possible that climbers may be causing damage to their limbs, both in the short term and in the long term [5, 12].

To our knowledge, this is the first description of the hot ache phenomenon in the medical literature. This study only sampled climbers from an online forum, and we acknowledge that it is possible that this introduced a selection bias, for example, age and internet access. The data for this study is self-reported and therefore may also be less robust. We assume that climbers know what the hot aches are and are thus able to describe what they have experienced. Future work should attempt to re-create the hot aches in a controlled environment. This will allow monitoring of physiological changes and the identification of biological changes that occur. It will also permit vascular dysfunction to be detected acutely or assessed in people who frequently experience the hot aches.

## Conclusions

The hot aches are a highly predictable and consistent experience for almost all winter climbers. This study has characterised, for the first time, a well recognised but previously unreported phenomenon that occurs in extreme winter climbers, with alarming frequency. The short- and long-term consequences of which are currently unknown and warrant further investigation.

## Additional file

Additional file 1 Raw Data. (XLSX 73 kb)

## Abbreviations

CIVD: Cold-induced vasodilatation
GTN: Glyceryl trinitrate
NFCI: Nonfreezing cold injury
NO: Nitric oxide
